# *In vivo* human coronary magnetic resonance angiography at 7 Tesla

**DOI:** 10.1186/1532-429X-11-S1-O46

**Published:** 2009-01-28

**Authors:** Saskia GC van Elderen, Andrew G Webb, Maarten Versluis, Jos Westenberg, Joost Doornbos, Nadine B Smith, Albert de Roos, Matthias Stuber

**Affiliations:** 1 LUMC, Leiden, Netherlands; 2 John Hopkins University, Baltimore, MD USA

**Keywords:** Right Coronary Artery, Specific Absorption Rate, Coronary Magnetic Resonance Angiography, Coronary MRAs, Radio Frequency Excitation

## Introduction

Coronary magnetic resonance angiography (MRA) is a promising technique for the non-invasive visualization of the coronary anatomy. However, due to the small dimensions and tortuous nature of the coronary arteries, high spatial resolution and volumetric coverage are mandatory. This requirement is critically linked with prolonged scanning times. The use of a high magnetic field strength has several potential advantages since the higher signal-to-noise ratio (SNR) may support improved spatial resolution and/or shortened scanning times. For these reasons we tested the hypotheses that in vivo human coronary MRA technology is feasible and can be implemented on a commercial 7 Tesla (T) system.

## Methods

Eight healthy volunteers (6 men, mean age 34 years ± 8) were positioned in a 7 T MR system (Philips Healthcare, Best, NL). A 13-cm diameter anterior surface coil was designed and constructed and used in transmit/receive mode. The size was chosen to achieve adequate radio frequency (RF) penetration. Commercial vector ECG (VECG) technology was used for R-wave triggering. Volume selective RF power optimization and shimming were applied for each scan. A segmented k-space gradient echo sequence was used for scout scanning. Multi-slice cine scans were used for coronary artery localization and for the visual identification of the time period (Td) of minimal coronary motion. Scan plane localization parallel to the right coronary artery (RCA) was facilitated using a three-point planscan tool. Double-oblique free-breathing 3D coronary MRA (segmented k-space gradient-echo imaging, TR = 4 ms, TE = 1.5 ms, RF excitation angle = 15°, field-of-view = 320 × 291 mm^2^, scan matrix = 392 × 373, 15 slices, slice thickness = 2 mm, acquisition window~100 ms, scan time~5 min) was performed using prospective navigator gating with the 2D selective navigator localized at the heart-lung interface. Image data were collected in mid-diastole at the predetermined Td. An adiabatic spectrally selective inversion recovery pre-pulse (TI = 200 ms) was used for fat suppression and enhanced contrast between the coronary blood-pool and epicardial fat. Coronary MRAs were reformatted and length measurements were performed using the "Soapbubble" software tool.

## Results

**Figure 1:**
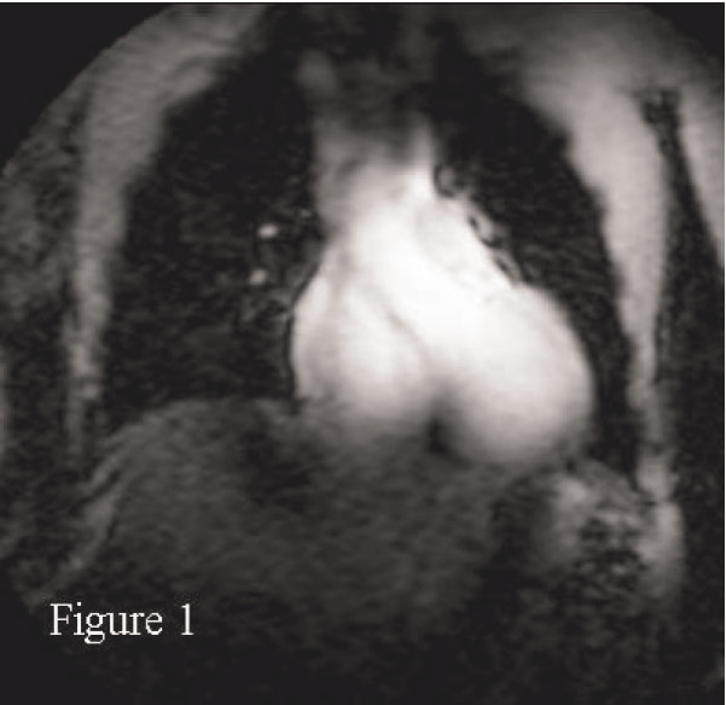
Figure 1

**Figure 2:**
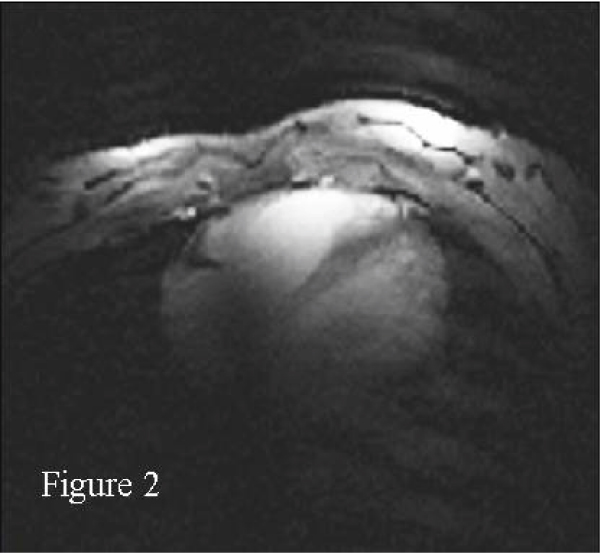
Figure 2

**Figure 3:**
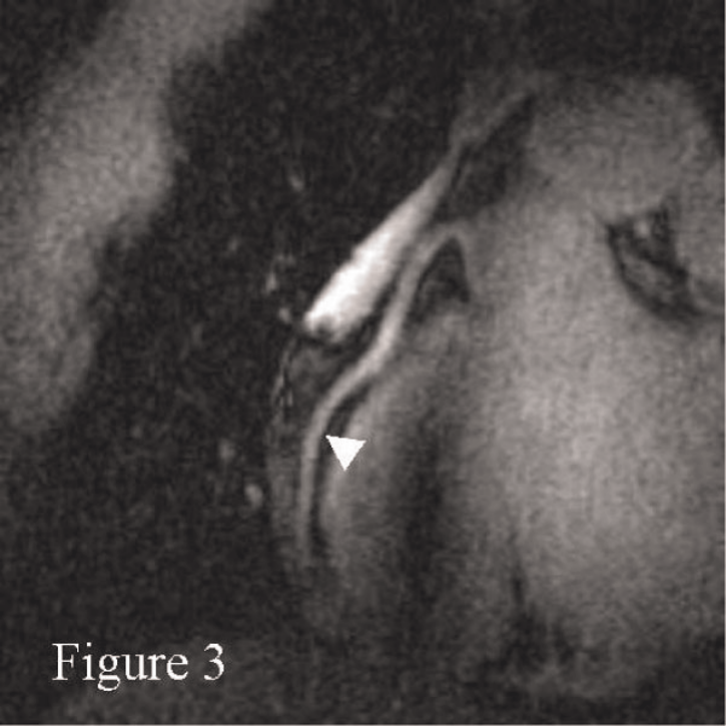
Figure 3

**Figure 4:**
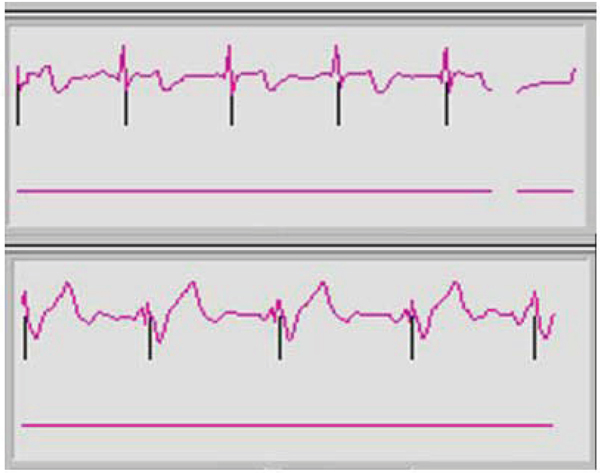
Figure 4 **(a) ECG outside the magnet; (b) ECG inside the magnet**.

**Figure 5:**
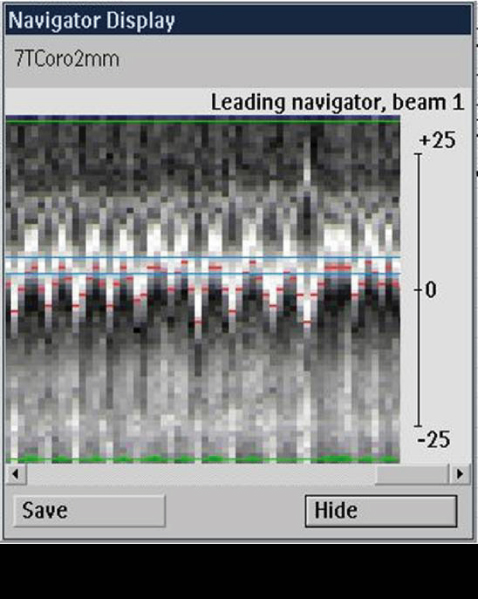
Figure 5

Right coronary MRAs were successfully obtained in all 8 healthy adult human subjects. Figures 1 and 2 show scout images in the coronal and axial plane, respectively, illustrating that the RF penetration and signal uniformity is sufficient for RCA imaging. Figure 3 shows a long contiguous segment of the RCA with high contrast between the blood-pool and the epicardial fat. The average measured contiguous length of the RCA was 77 ± 35 mm. One potential concern is that, at the higher field strength, the magneto-hydrodynamic effect is amplified with an artificial augmentation of the T-wave of the ECG (Figure 4a and 4b). Nevertheless, the VECG algorithm allowed reliable R-wave triggering. In Figure 5 the navigator signal from the heart-lung interface received by the surface coil can be seen.

## Discussion

To our knowledge, this is the first report of human coronary MRA at 7 T. With suitable adaptations of the scanning protocol (e.g. navigator localization and use of a spectrally selective adiabatic inversion recovery for fat saturation) and the use of a custom-built transmit-receive surface coil, coronary MRA technology has been successfully implemented at 7 T and long contiguous segments of the RCA can be obtained in vivo and in humans. Conventional T2-weighted preparation cannot currently be used for contrast generation because of conservative specific absorption rate (SAR) constraints. Future work will focus on optimizing contrast enhancement between the blood-pool and the myocardium within the SAR and B1 homogeneity constraints. In order to improve volumetric coverage, the development of larger surface coils or coil arrays will be required. The latter will be most important in the trade-off between the expected SNR benefit vs. shorter scanning times.

In conclusion, navigator gated free-breathing 3D coronary MRA has successfully been implemented in vivo and in humans on a commercial 7 T system.

